# 7-Chloro-3-phenyl­benzo[4,5]thia­zolo[2,3-*c*][1,2,4]triazole

**DOI:** 10.1107/S1600536811039456

**Published:** 2011-09-30

**Authors:** Hoong-Kun Fun, Safra Izuani Jama Asik, M. Himaja, D. Munirajasekhar, B. K. Sarojini

**Affiliations:** a X-ray Crystallography Unit, School of Physics, Universiti Sains Malaysia, 11800 USM, Penang, Malaysia; b Chemistry Division, School of Advanced Sciences, VIT University, Vellore-632014, Tamil Nadu, India; c Department of Chemistry, P A College of Engineering, Nadupadavu, D.K., Mangalore, India

## Abstract

In the title compound, C_14_H_8_ClN_3_S, the dihedral angle between the approximately planar triple-fused ring system (r.m.s. deviation = 0.065 Å) and the pendant phenyl ring is 62.25 (5)°. In the crystal, mol­ecules are linked into infinite chains along the *c*-axis direction by C—H⋯N hydrogen bonds. Aromatic π–π stacking inter­actions [centroid–centroid distances = 3.7499 (8) and 3.5644 (8) Å] and weak C—H⋯π inter­actions are also observed.

## Related literature

For the biological activity of benzothio­zole derivatives, see: Yaseen *et al.* (2006 ▶); Kini *et al.* (2007 ▶); Munirajasekhar *et al.* (2011 ▶); Gurupadayya *et al.* (2008 ▶); Bowyer *et al.* (2007 ▶); Mittal *et al.* (2007 ▶); Pozas *et al.* (2005 ▶); Rana *et al.* (2008 ▶).
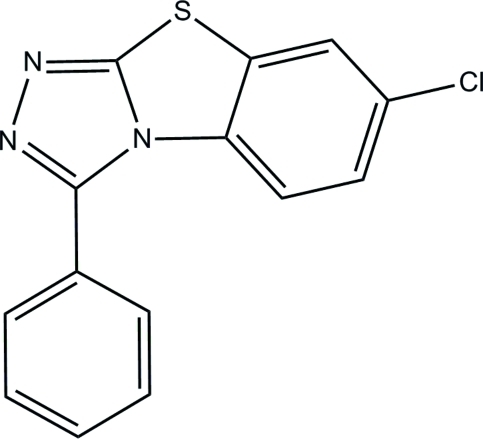


## Experimental

### 

#### Crystal data

C_14_H_8_ClN_3_S
*M* *_r_* = 285.74
Monoclinic, 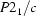

*a* = 16.9941 (13) Å
*b* = 5.8895 (5) Å
*c* = 12.0930 (9) Å
β = 91.770 (1)°
*V* = 1209.77 (16) Å^3^
*Z* = 4
Mo *K*α radiation
μ = 0.47 mm^−1^
*T* = 296 K
0.41 × 0.31 × 0.18 mm

#### Data collection

Bruker APEX DUO CCD diffractometer
Absorption correction: multi-scan (*SADABS*; Bruker, 2009 ▶) *T*_min_ = 0.828, *T*_max_ = 0.919
14981 measured reflections
4033 independent reflections
3342 reflections with *I* > 2σ(*I*)
*R*_int_ = 0.021

#### Refinement

*R*[*F* ^2^ > 2σ(*F* ^2^)] = 0.033
*wR*(*F* ^2^) = 0.101
*S* = 1.02
4033 reflections
172 parameters
H-atom parameters constrained
Δρ_max_ = 0.30 e Å^−3^
Δρ_min_ = −0.23 e Å^−3^

### 

Data collection: *APEX2* (Bruker, 2009 ▶); cell refinement: *SAINT* (Bruker, 2009 ▶); data reduction: *SAINT*; program(s) used to solve structure: *SHELXTL* (Sheldrick, 2008 ▶); program(s) used to refine structure: *SHELXTL*; molecular graphics: *SHELXTL*; software used to prepare material for publication: *SHELXTL* and *PLATON* (Spek, 2009 ▶).

## Supplementary Material

Crystal structure: contains datablock(s) global, I. DOI: 10.1107/S1600536811039456/hb6414sup1.cif

Structure factors: contains datablock(s) I. DOI: 10.1107/S1600536811039456/hb6414Isup2.hkl

Supplementary material file. DOI: 10.1107/S1600536811039456/hb6414Isup3.cml

Additional supplementary materials:  crystallographic information; 3D view; checkCIF report

## Figures and Tables

**Table 1:** Hydrogen-bond geometry (Å, °) *Cg*3 is the centroid of the C1–C6 ring.

*D*—H⋯*A*	*D*—H	H⋯*A*	*D*⋯*A*	*D*—H⋯*A*
C10—H10*A*⋯N3^i^	0.93	2.57	3.3135 (16)	138
C4—H4*A*⋯*Cg*3^ii^	0.93	2.92	3.5851 (15)	130

Symmetry codes: (i) 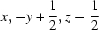; (ii) 
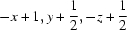
.

**Fig. 1.**
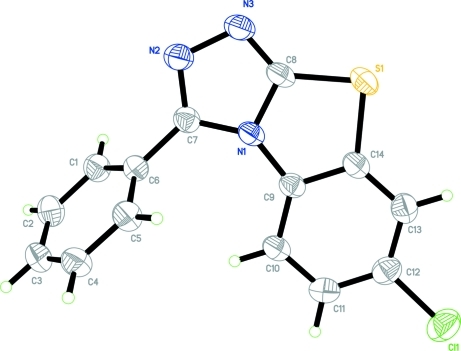
The structure of the title compound, showing 50% probability displacement ellipsoids. Hydrogen atoms are shown as spheres of arbitrary radius.

**Fig. 2.**
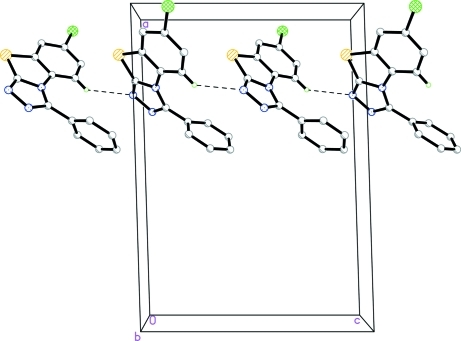
The crystal packing, viewed along the b-axis, showing the molecules linked into infinite chains along the c axis. Hydrogen atoms that not involved in hydrogen bonding (dashed lines) are omitted for clarity.

## supplementary crystallographic information

### Comment

Benzothiazole derivatives have emerged as significant components in various diversified therapeutic applications. Literature review reveals that benzothiazoles and their derivatives show considerable activity including potent inhibition of human immunodeficiency virus type 1 (HIV-1), replication by HIV-1 protease inhibition (Yaseen *et al.*,2006), antitumor (Kini *et al.*, 2007), anthelmintic (Munirajasekhar *et al.*, 2011), analgesic, anti-inflammatory (Gurupadayya *et al.*,2008), antimalarial (Bowyer *et al.*,2007), antifungal (Mittal *et al.*, 2007), anticandidous (Pozas *et al.*,2005) and various CNS activities (Rana *et al.*, 2008). The present work describes the synthesis and crystal structure of the title compound, 7-Chloro-3- phenylbenzo[4,5]thiazolo[2,3-*c*][1,2,4]triazole, which was prepared from the reaction of 2-benzylidene-1-(6-chlorobenzo[d]thiazol-2-yl)hydrazine treated  with  iodobenzene diacetate.

In the title compound of (I), (Fig. 1), the benzene (C9–C14) ring makes dihedral angles of 5.59 (7) and 2.45 (6)° with the thiazole ring (S1/N1/C8/C9/C14) and the mean plane of triazole (N1–N3/C7/C8) ring, respectively. The dihedral angle between the two benzene (C1–C6 and C9–C14) rings is 64.11 (6)°.

In the crystal structure of (Fig. 2), the molecules are linked into infinite chains along the *c* axis by C10—H10A···N3 hydrogen bonds. π–π stacking interactions are observed between the triazole (N1–N3/C7/C8) ; centroid *Cg*2) and benzene (C1–C6) ; centroid *Cg*3) rings with a distance of *Cg*2···*Cg*3 = 3.7499 (8) Å and between triazole (N1–N3/C7/C8) ; centroid *Cg*2) and benzene (C9–C14) ; centroid *Cg*4) rings with a separation of *Cg*2···*Cg*4 = 3.5644 (8) Å. Furthermore the crystal structure is stabilized by weak C—H···π interactions (Table 1) with distance of 3.5851 (15) Å.

### Experimental

To a solution of the 2-benzylidene-1-(6-chlorobenzo[d]thiazol-2-yl)hydrazine (2 mmol) in dichloromethane (10 mL) at room temperature, iodobenzene diacetate (2 mmol) was added in 2–3 portions over 5 min. The resultant reaction mixture was stirred for 45 min. The solvent was evaporated under high vacuum and then purified by column chromatography (40% ethyl acetate in chloroform). The product was recrystalized from ethanol to give colourless blocks.

### Refinement

All the H atoms were placed in calculated positions with C–H = 0.93 Å. The *U*_iso_ values were constrained to be 1.2*U*_eq_ of the carrier atom for the H atoms.

### Crystal data

**Table:**

C_14_H_8_ClN_3_S	*F*(000) = 584
*M**_r_* = 285.74	*D*_x_ = 1.569 Mg m^−^^3^
Monoclinic, *P*2_1_/*c*	Mo *K*α radiation, λ = 0.71073 Å
Hall symbol: -P 2ybc	Cell parameters from 6868 reflections
*a* = 16.9941 (13) Å	θ = 2.4–31.4°
*b* = 5.8895 (5) Å	µ = 0.47 mm^−^^1^
*c* = 12.0930 (9) Å	*T* = 296 K
β = 91.770 (1)°	Block, colourless
*V* = 1209.77 (16) Å^3^	0.41 × 0.31 × 0.18 mm
*Z* = 4	

### Data collection

**Table:**

Bruker APEX DUO CCD diffractometer	4033 independent reflections
Radiation source: fine-focus sealed tube	3342 reflections with *I* > 2σ(*I*)
graphite	*R*_int_ = 0.021
φ and ω scans	θ_max_ = 31.7°, θ_min_ = 2.4°
Absorption correction: multi-scan (*SADABS*; Bruker, 2009)	*h* = −23→25
*T*_min_ = 0.828, *T*_max_ = 0.919	*k* = −8→6
14981 measured reflections	*l* = −17→17

### Refinement

**Table:**

Refinement on *F*^2^	Primary atom site location: structure-invariant direct methods
Least-squares matrix: full	Secondary atom site location: difference Fourier map
*R*[*F*^2^ > 2σ(*F*^2^)] = 0.033	Hydrogen site location: inferred from neighbouring sites
*wR*(*F*^2^) = 0.101	H-atom parameters constrained
*S* = 1.02	*w* = 1/[σ^2^(*F*_o_^2^) + (0.051*P*)^2^ + 0.2986*P*] where *P* = (*F*_o_^2^ + 2*F*_c_^2^)/3
4033 reflections	(Δ/σ)_max_ < 0.001
172 parameters	Δρ_max_ = 0.30 e Å^−^^3^
0 restraints	Δρ_min_ = −0.23 e Å^−^^3^

### Special details

**Table:**

Geometry. All esds (except the esd in the dihedral angle between two l.s. planes) are estimated using the full covariance matrix. The cell esds are taken into account individually in the estimation of esds in distances, angles and torsion angles; correlations between esds in cell parameters are only used when they are defined by crystal symmetry. An approximate (isotropic) treatment of cell esds is used for estimating esds involving l.s. planes.
Refinement. Refinement of F^2^ against ALL reflections. The weighted R-factor wR and goodness of fit S are based on F^2^, conventional R-factors R are based on F, with F set to zero for negative F^2^. The threshold expression of F^2^ > 2sigma(F^2^) is used only for calculating R-factors(gt) etc. and is not relevant to the choice of reflections for refinement. R-factors based on F^2^ are statistically about twice as large as those based on F, and R- factors based on ALL data will be even larger.

### Fractional atomic coordinates and isotropic or equivalent isotropic displacement parameters (Å^2^)

**Table:**

	*x*	*y*	*z*	*U*_iso_*/*U*_eq_	
S1	0.14776 (2)	0.26324 (6)	0.56813 (2)	0.04078 (10)	
Cl1	0.03783 (2)	1.00056 (7)	0.32558 (4)	0.05570 (12)	
N1	0.24962 (6)	0.23101 (16)	0.41312 (8)	0.03099 (19)	
N2	0.31988 (7)	−0.07810 (19)	0.44194 (8)	0.0384 (2)	
N3	0.26554 (7)	−0.06213 (19)	0.52726 (9)	0.0401 (2)	
C1	0.35771 (8)	−0.0067 (2)	0.19026 (10)	0.0383 (3)	
H1A	0.3299	−0.1423	0.1940	0.046*	
C2	0.40171 (9)	0.0410 (3)	0.09818 (11)	0.0454 (3)	
H2A	0.4029	−0.0621	0.0400	0.055*	
C3	0.44371 (8)	0.2413 (3)	0.09278 (12)	0.0455 (3)	
H3A	0.4736	0.2719	0.0314	0.055*	
C4	0.44144 (8)	0.3969 (2)	0.17883 (11)	0.0430 (3)	
H4A	0.4698	0.5316	0.1750	0.052*	
C5	0.39691 (7)	0.3515 (2)	0.27057 (10)	0.0371 (2)	
H5A	0.3948	0.4566	0.3278	0.045*	
C6	0.35531 (6)	0.1482 (2)	0.27673 (9)	0.0309 (2)	
C7	0.30952 (7)	0.0965 (2)	0.37544 (9)	0.0316 (2)	
C8	0.22587 (7)	0.1233 (2)	0.50709 (9)	0.0344 (2)	
C9	0.20460 (6)	0.42020 (19)	0.38183 (9)	0.0297 (2)	
C10	0.21108 (7)	0.5535 (2)	0.28811 (9)	0.0340 (2)	
H10A	0.2495	0.5239	0.2369	0.041*	
C11	0.15893 (8)	0.7317 (2)	0.27264 (11)	0.0382 (3)	
H11A	0.1620	0.8234	0.2103	0.046*	
C12	0.10195 (7)	0.7744 (2)	0.34998 (11)	0.0383 (3)	
C13	0.09432 (7)	0.6425 (2)	0.44419 (10)	0.0386 (3)	
H13A	0.0559	0.6734	0.4952	0.046*	
C14	0.14623 (7)	0.4629 (2)	0.45909 (9)	0.0334 (2)	

### Atomic displacement parameters (Å^2^)

**Table:**

	*U*^11^	*U*^22^	*U*^33^	*U*^12^	*U*^13^	*U*^23^
S1	0.04819 (18)	0.04410 (18)	0.03082 (15)	−0.00110 (13)	0.01342 (12)	0.00439 (12)
Cl1	0.0502 (2)	0.0465 (2)	0.0703 (3)	0.01117 (15)	−0.00102 (16)	0.00577 (17)
N1	0.0376 (5)	0.0308 (5)	0.0248 (4)	−0.0036 (4)	0.0054 (3)	0.0017 (3)
N2	0.0467 (5)	0.0359 (5)	0.0328 (5)	0.0011 (4)	0.0035 (4)	0.0031 (4)
N3	0.0516 (6)	0.0377 (5)	0.0313 (5)	−0.0006 (5)	0.0058 (4)	0.0061 (4)
C1	0.0435 (6)	0.0336 (6)	0.0379 (6)	−0.0007 (5)	0.0065 (5)	−0.0041 (5)
C2	0.0540 (7)	0.0473 (7)	0.0357 (6)	0.0068 (6)	0.0112 (5)	−0.0061 (5)
C3	0.0442 (7)	0.0528 (8)	0.0403 (6)	0.0066 (6)	0.0146 (5)	0.0078 (6)
C4	0.0399 (6)	0.0421 (7)	0.0475 (7)	−0.0046 (5)	0.0072 (5)	0.0075 (5)
C5	0.0394 (6)	0.0360 (6)	0.0362 (6)	−0.0041 (5)	0.0037 (4)	−0.0035 (5)
C6	0.0319 (5)	0.0317 (5)	0.0292 (5)	0.0004 (4)	0.0026 (4)	0.0001 (4)
C7	0.0361 (5)	0.0306 (5)	0.0283 (5)	−0.0018 (4)	0.0027 (4)	−0.0017 (4)
C8	0.0436 (6)	0.0353 (6)	0.0246 (5)	−0.0056 (5)	0.0054 (4)	0.0032 (4)
C9	0.0342 (5)	0.0289 (5)	0.0261 (4)	−0.0042 (4)	0.0022 (4)	−0.0007 (4)
C10	0.0384 (5)	0.0351 (6)	0.0288 (5)	−0.0050 (4)	0.0040 (4)	0.0021 (4)
C11	0.0412 (6)	0.0370 (6)	0.0361 (6)	−0.0036 (5)	−0.0017 (4)	0.0066 (5)
C12	0.0364 (5)	0.0334 (6)	0.0449 (6)	−0.0014 (4)	−0.0027 (5)	0.0004 (5)
C13	0.0367 (6)	0.0395 (6)	0.0398 (6)	−0.0018 (5)	0.0061 (4)	−0.0038 (5)
C14	0.0373 (5)	0.0343 (5)	0.0288 (5)	−0.0049 (4)	0.0054 (4)	−0.0003 (4)

### Geometric parameters (Å, °)

**Table:**

S1—C8	1.7454 (13)	C3—H3A	0.9300
S1—C14	1.7663 (12)	C4—C5	1.3881 (17)
Cl1—C12	1.7405 (13)	C4—H4A	0.9300
N1—C8	1.3729 (13)	C5—C6	1.3935 (16)
N1—C7	1.3783 (15)	C5—H5A	0.9300
N1—C9	1.3972 (14)	C6—C7	1.4768 (15)
N2—C7	1.3140 (16)	C9—C10	1.3860 (15)
N2—N3	1.4088 (15)	C9—C14	1.4062 (15)
N3—C8	1.3026 (17)	C10—C11	1.3826 (17)
C1—C6	1.3895 (16)	C10—H10A	0.9300
C1—C2	1.3891 (18)	C11—C12	1.3898 (19)
C1—H1A	0.9300	C11—H11A	0.9300
C2—C3	1.381 (2)	C12—C13	1.3882 (18)
C2—H2A	0.9300	C13—C14	1.3856 (17)
C3—C4	1.388 (2)	C13—H13A	0.9300
			
C8—S1—C14	89.57 (5)	N2—C7—N1	109.50 (10)
C8—N1—C7	104.31 (10)	N2—C7—C6	126.31 (11)
C8—N1—C9	114.84 (10)	N1—C7—C6	124.17 (10)
C7—N1—C9	140.59 (9)	N3—C8—N1	112.25 (10)
C7—N2—N3	108.51 (10)	N3—C8—S1	135.45 (9)
C8—N3—N2	105.43 (10)	N1—C8—S1	112.26 (9)
C6—C1—C2	119.94 (12)	C10—C9—N1	128.07 (10)
C6—C1—H1A	120.0	C10—C9—C14	121.17 (11)
C2—C1—H1A	120.0	N1—C9—C14	110.75 (10)
C3—C2—C1	120.18 (13)	C11—C10—C9	118.29 (11)
C3—C2—H2A	119.9	C11—C10—H10A	120.9
C1—C2—H2A	119.9	C9—C10—H10A	120.9
C2—C3—C4	120.13 (12)	C10—C11—C12	120.20 (12)
C2—C3—H3A	119.9	C10—C11—H11A	119.9
C4—C3—H3A	119.9	C12—C11—H11A	119.9
C3—C4—C5	120.03 (13)	C11—C12—C13	122.41 (12)
C3—C4—H4A	120.0	C11—C12—Cl1	118.02 (10)
C5—C4—H4A	120.0	C13—C12—Cl1	119.57 (10)
C4—C5—C6	119.88 (12)	C14—C13—C12	117.30 (11)
C4—C5—H5A	120.1	C14—C13—H13A	121.3
C6—C5—H5A	120.1	C12—C13—H13A	121.3
C1—C6—C5	119.83 (10)	C13—C14—C9	120.62 (11)
C1—C6—C7	120.08 (11)	C13—C14—S1	126.87 (9)
C5—C6—C7	120.09 (10)	C9—C14—S1	112.50 (9)
			
C7—N2—N3—C8	−0.41 (14)	C7—N1—C8—S1	−178.75 (8)
C6—C1—C2—C3	0.6 (2)	C9—N1—C8—S1	−3.47 (13)
C1—C2—C3—C4	−0.7 (2)	C14—S1—C8—N3	−175.12 (14)
C2—C3—C4—C5	0.0 (2)	C14—S1—C8—N1	2.37 (9)
C3—C4—C5—C6	0.8 (2)	C8—N1—C9—C10	−175.66 (11)
C2—C1—C6—C5	0.19 (19)	C7—N1—C9—C10	−2.9 (2)
C2—C1—C6—C7	−179.13 (12)	C8—N1—C9—C14	2.82 (14)
C4—C5—C6—C1	−0.91 (18)	C7—N1—C9—C14	175.61 (13)
C4—C5—C6—C7	178.41 (11)	N1—C9—C10—C11	178.84 (11)
N3—N2—C7—N1	0.01 (13)	C14—C9—C10—C11	0.51 (17)
N3—N2—C7—C6	178.56 (11)	C9—C10—C11—C12	0.20 (18)
C8—N1—C7—N2	0.37 (13)	C10—C11—C12—C13	−0.4 (2)
C9—N1—C7—N2	−172.89 (13)	C10—C11—C12—Cl1	−179.79 (9)
C8—N1—C7—C6	−178.22 (10)	C11—C12—C13—C14	−0.12 (19)
C9—N1—C7—C6	8.5 (2)	Cl1—C12—C13—C14	179.26 (9)
C1—C6—C7—N2	59.23 (17)	C12—C13—C14—C9	0.83 (17)
C5—C6—C7—N2	−120.09 (14)	C12—C13—C14—S1	−177.69 (9)
C1—C6—C7—N1	−122.43 (13)	C10—C9—C14—C13	−1.05 (17)
C5—C6—C7—N1	58.25 (16)	N1—C9—C14—C13	−179.65 (10)
N2—N3—C8—N1	0.66 (14)	C10—C9—C14—S1	177.67 (9)
N2—N3—C8—S1	178.15 (11)	N1—C9—C14—S1	−0.93 (12)
C7—N1—C8—N3	−0.66 (13)	C8—S1—C14—C13	177.83 (12)
C9—N1—C8—N3	174.63 (10)	C8—S1—C14—C9	−0.79 (9)

### Hydrogen-bond geometry (Å, °)

**Table:**

Cg3 is the centroid of the C1–C6 ring.

**Table:**

*D*—H···*A*	*D*—H	H···*A*	*D*···*A*	*D*—H···*A*
C10—H10A···N3^i^	0.93	2.57	3.3135 (16)	138
C4—H4A···Cg3^ii^	0.93	2.92	3.5851 (15)	130

Symmetry codes: (i) *x*, −*y*+1/2, *z*−1/2; (ii) −*x*+1, *y*+1/2, −*z*+1/2.

## References

[bb1] Bowyer, P.W., Ruwani, S & Gunaratne. (2007). *Biochem J.* **2**, 173–180. 10.1042/BJ20070692 PMC2267354 17714074

[bb2] Bruker (2009). *APEX2*, *SAINT* and *SADABS* Bruker AXS Inc., Madison, Wisconsin, USA.

[bb3] Gurupadayya, B. M., Gopal, M., Padmashali, B. & Manohara, Y. N. (2008). *Indian J. Pharm. Sci.* **70**, 572–577. 10.4103/0250-474X.45393 PMC3038279 21394251

[bb4] Kini, S., Swain, S. P. & Gandhi, A. M. (2007). *Indian J. Pharm. Sci.* **69**, 46–50.

[bb5] Mittal, S., Samottra, M. K., Kaur, J. & Gita, S. (2007). *Phosphorus*, *Sulfur Silicon Relat. Elem.* **9**, 2105–2113.

[bb6] Munirajasekhar, D., Himaja, M. & Sunil, V. M. (2011). *Intl Res. J. Pharm.* **2**, 114–117.

[bb7] Pozas, R., Carballo, J., Castro, C. & Rubio, J. (2005). *Bioorg. Med. Chem. Lett.* **15**, 1417–421. 10.1016/j.bmcl.2005.01.002 15713399

[bb8] Rana, A., Siddiqui, N. & Khan, S. (2008). *Eur. J. Med. Chem.* **43**, 1114–1122. 10.1016/j.ejmech.2007.07.008 17826870

[bb9] Sheldrick, G. M. (2008). *Acta Cryst.* A**64**, 112–122. 10.1107/S0108767307043930 18156677

[bb10] Spek, A. L. (2009). *Acta Cryst.* D**65**, 148–155. 10.1107/S090744490804362X PMC2631630 19171970

[bb11] Yaseen, A., Haitham, A. S., Houssain, A. S. & Najim, A. (2006). *Z. Naturforsch.* **62**, 523–528.

